# Essentiality of Trace Elements in Pregnancy, Fertility, and Gynecologic Cancers—A State-of-the-Art Review

**DOI:** 10.3390/nu14010185

**Published:** 2021-12-31

**Authors:** James Curtis Dring, Alicja Forma, Zuzanna Chilimoniuk, Maciej Dobosz, Grzegorz Teresiński, Grzegorz Buszewicz, Jolanta Flieger, Tomasz Cywka, Jacek Januszewski, Jacek Baj

**Affiliations:** 1 Chair and Department of Forensic Medicine, Medical University of Lublin, ul. Jaczewskiego 8B, 20-090 Lublin, Poland; jcdring@gmail.com (J.C.D.); aforma@onet.pl (A.F.); zuzia.chil@gmail.com (Z.C.); macdob.98@gmail.com (M.D.); grzegorz.teresinski@umlub.pl (G.T.); grzegorz.buszewicz@umlub.pl (G.B.); tomasz.cywka@umlub.pl (T.C.); jacek.januszewski000@gmail.com (J.J.); 2 Department of Analytical Chemistry, Medical University of Lublin, ul. Chodzki 4A, 20-093 Lublin, Poland; j.flieger@umlub.pl; 3 Chair and Department of Anatomy, Medical University of Lublin, ul. Jaczewskiego 4, 20-090 Lublin, Poland

**Keywords:** trace metals, fertility, carcinogenesis, female reproductive system, gynecology

## Abstract

Gynecological neoplasms pose a serious threat to women’s health. It is estimated that in 2020, there were nearly 1.3 million new cases worldwide, from which almost 50% ended in death. The most commonly diagnosed are cervical and endometrial cancers; when it comes to infertility, it affects ~48.5 million couples worldwide and the number is continually rising. Ageing of the population, environmental factors such as dietary habits, environmental pollutants and increasing prevalence of risk factors may affect the reproductive potential in women. Therefore, in order to identify potential risk factors for these issues, attention has been drawn to trace elements. Trace mineral imbalances can be caused by a variety of causes, starting with hereditary diseases, finishing with an incorrect diet or exposure to polluted air or water. In this review, we aimed to summarize the current knowledge regarding trace elements imbalances in the case of gynecologic cancers as well as female fertility and during pregnancy.

## 1. Introduction

A woman’s age is the most important factor in determining her fertility potential, which decreases gradually from, approximately, the age of 32 to accelerated decline after the age of 37. Declining germ cells quantity and quality has the biggest impact on the change in fecundity [1,2,3]. The maximum number of oocytes (6–7 million) occurs at the 20th week of gestation in a female fetus, but after birth, the number gradually decreases from about 1–2 million to 300,000–500,000 in adolescence and 1000 in the average age of menopause with no further neogenesis [4,5]. This is extremely important because there has been a societal shift towards postponed childbirth leading to an increase in permanent involuntary childlessness [6,7].

Various environmental factors such as dietary habits, lifestyle, and environmental pollutants may affect the aging process and function of the human oocytes [8,9]. In addition, the control of other modifiable risk factors, including smoking, alcohol consumption, stress exposure, and obesity or low weight may contribute to maintaining oocyte quality [10,11].

Cancers constitute a leading cause of death worldwide both in high-income and low-income countries [12]. Gynecological neoplasms are considered the most common cancers among women worldwide, with cervical cancer as the second most common and endometrial as the sixth most prevalently diagnosed [13,14]. It is estimated that in 2020, there were 604,127 new cases and 341,000 deaths from cervical cancer, 417,367 new cases and 97,370 deaths from cancer of the corpus uteri, 313,959 new cases and 207,252 deaths from ovarian cancer, 45,240 new cases and 7427 deaths from vulvar cancer and 17,908 new cases and 7995 deaths from vaginal cancer [13]. Due to the ageing, growth of the population and increasing prevalence of risk factors, the burden associated with cancer incidence and mortality is rapidly expanding [13]. 

Trace elements (TE) are defined as minerals that are required in amounts of 1–100 mg/day by adults or account for extremely small quantities of less than 0.01% of the total body weight. Essential trace elements of the human body include zinc (Zn), copper (Cu), selenium (Se), chromium (Cr), cobalt (Co), iodine (I), manganese (Mn) and molybdenum (Mo) [15,16,17]. TE as components of complexes are essential in fundamental metabolic processes such as enzymatic reactions and also play significant roles in functioning of proteins and transcriptional factors [18,19,20].

Trace mineral imbalance may be caused by hereditary disorders, kidney dialysis, parenteral nutrition, restrictive diets or dietary patterns [18]. As a result, micronutrient deficits may lead to the dysregulation of cellular function as the consequence of the functional enzyme disorder [21]. 

Deficiency of some trace elements may also have a negative influence on reproductive function and sperm quality, being a significant factor for impaired spermatogenesis [22]. Additionally, a link between several trace elements concentration and both time to pregnancy (TTP) and subfertility was identified. It has been reported that lower plasma zinc and selenium concentrations in pregnant women were associated with longer TTP, while lower selenium concentrations were considered as a great risk factor for subfertility [23,24].

However, numerous publications have addressed the problem of TE intoxication, suggesting that manganese exposures may occur in nutritional sources, contaminated foods, water, soil, and air with contaminations. As a result, Mn exposure disrupted in neurogenesis, impaired dopaminergic, glutamatergic, and GABAergic transmission with clinical signs and symptoms resembling Parkinson’s disease [25,26]. Additionally, accumulation of chromium in edible parts of plants in higher concentrations than the maximum permitted limits can cause numerous health risks for consumers, including clinical disorders such as respiratory, carcinogenic, renal, hepatic, gastrointestinal, cardiovascular ones, etc. [27,28]. In addition, several studies associated occupational exposure to Cr with increased risk of respiratory system cancers, especially lung, nasal, and sinus cancers, at the same time pointing to Cr as the agent in carcinogenesis [29,30]. Lin and Yang’s meta-analysis and Mendelian randomization study found a link between lower circulating zinc levels and a higher risk of ovarian cancer. However, there was no causal effect of circulating copper on ovarian cancer risk [31]. In another study, high serum selenium concentration was associated with cervical cancer. Due to its increased levels after the treatment, selenium was indicated as a protective factor for cervical cancer [32].

The aim of this review was to present the current knowledge regarding the fluctuations in trace elements concentrations during pregnancy as well as in the case of gynaecologic cancers. We also aimed to present the alterations in trace elements concentrations with regards to female fertility.

## 2. Female Infertility

According to the current definition, “infertility” is a disease characterized by the failure of establishing a clinical pregnancy after 12 months of regular, unprotected sexual intercourse or due to an impairment of a person’s capacity to reproduce, either as an individual or with his/her partner. Infertility can be further classified as primary when a woman has never been clinically pregnant or secondary when a woman has been pregnant at least once before. The idea of infertility applies only to a limited period of time because the permanent state of infertility is called sterility [33].

It has been estimated that infertility affects ~48.5 million (45.0 million, 52.6 million) couples worldwide. In 2010, it was found that 1.9% of women aged 20–44 years struggled with primary infertility, and 10.5% of women struggled with secondary infertility. The highest prevalence of infertility was observed in regions such as South Asia, some countries of sub-Saharan Africa, the Middle East, North Africa, Central and Eastern Europe, and Central Asia. The lowest rates were noted in Western countries [34].

There are combinations of many factors that can preclude conception. Most of them can be divided into three categories, related to genital etiology, extragenital etiology, and those caused by psychological or environmental factors [35,36]. It is also worth mentioning that in 8–20% of cases, the main cause of infertility is unexplained [37].

**Figure 1:**
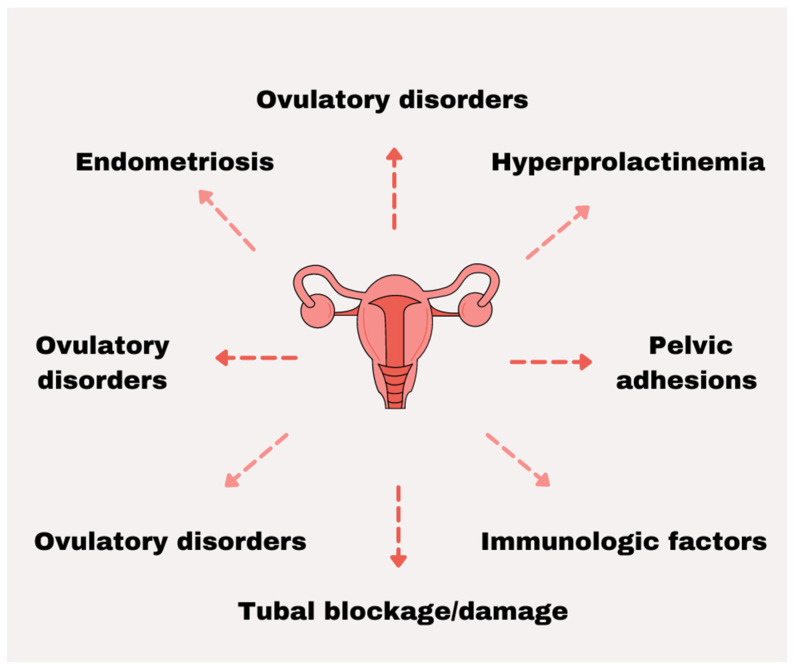
The most prevalent factors associated with female infertility.

The function of the female reproductive organs may be impaired by congenital or acquired defects [35]. Ovarian disorders are often caused by polycystic ovary syndrome (POS), premature ovarian insufficiency (POI), or other follicular disorders. Incorrect condition of fallopian tubes occurs as a result of lack of patency and mobility which are most often caused by untreated sexually transmitted infections, sepsis, or complications of unsafe abortion or abdominal surgery. The main causes of uterine disorders are endometriosis, uterine fibroids, or endometrial polyps [38]. Nongenital illnesses can also result in invalid conception. Hypogonadotropic hypogonadism or hyperprolactinemia leads to imbalances in reproductive hormones [39]. It has been reported that some systemic illnesses such as sepsis, severe renal disease, unstable diabetes, celiac disease, or autoimmune thyroiditis may negatively affect the fertility of women. Epidemiological studies suggest that the environment and lifestyle of a woman can influence her fertility [39,40,41,42]. Stress, obesity, excessive smoking, and alcohol intake are believed to contribute to recurrent implantation failure and difficulties in conceiving [39,43] (Figure 1).

## 3. Most Prevalent Cancers of the Female Reproductive System

### 3.1. Cervical Cancer

Cervical cancer is the most common genital cancer. According to the latest GLOBOCAN 2020, during the last year, 604,127 new cases of cervical cancer were diagnosed, ranking it eighth among all cancers and fourth among female cancers, worldwide. Cervical cancer is ninth for leading causes of cancer death, causing more than 341,000 deaths per year, in both sexes together. The highest incidence rates of cervical cancer are found in Eastern and Southern Africa and the lowest in Western Asia, Australia and New Zealand, and Northern America [13].

Infection with human papillomavirus (HPV) is a factor that initiates the process of carcinogenesis. HPV types 16 and 18 are present in 70% cases [44,45]. The virus is transmitted between partners during sexual intercourse, hand to genital organ contact, or oral sex. The infection spreads very easily and is the most common sexually transmitted infection in the world [46]. Risks factors for HPV and cervical cancer include early age of first sexual intercourse, multiple sexual partners, smoking, herpes simplex or HIV infection, hampered access to screening programs, and oral contraceptive use [47,48,49,50].

In the early stages, cervical cancer is usually asymptomatic. Later symptoms may include abnormal vaginal bleeding, profuse malodorous vaginal discharge, or pelvic pain [51].

Primary prevention of HPV infection relies on avoiding risky sexual activity and using condoms. Additional effective strategies in cervical cancer prevention contain HPV screening and vaccination programs. Studies have shown that the estimated effectiveness of vaccination against HPV 16 and 18 is more than 90% [45,52]. In countries where at least 50% of women were vaccinated, HPV types 16 and 18 infections were significantly reduced by 68% [52].

### 3.2. Ovarian Cancer

Ovarian cancer is the most fatal genital cancer [53,54]. According to current data, it is estimated that in 2020, 313,959 new cases and 207,252 deaths occurred worldwide [13]. This cancer is very rare in women under the age of 40. Above 50 years of age, the risk increases drastically, and the average diagnosis is between 50 and 70 years of age [55]. Studies have shown that the median ovarian cancer survival rate after five years is ~49.1%. In the late stage, this figure reaches 29%, while it is 92% in the early stage of the disease [56].

High mortality numbers are caused by unclear symptoms, late diagnosis, and frequent recurrence. Most patients are diagnosed with stage III or IV of the disease, which makes treatment difficult [57]. Most women report nonspecific symptoms such as bloating and abdominal pain, frequent urination, early feeling of fullness, and changes in bowel habits. As a result, these symptoms are often overlooked, and further diagnostics are not implemented [53].

Modifiable risk factors for cancer include smoking, hormonal replacement therapy, and dietary factors such as low fiber or vitamin D deficiency [58]. Family history of ovarian or breast cancer greatly increases the risk of developing ovarian cancer. It is also associated with the presence of mutations in the BRCA1, BRCA2, and MMR genes, which may increase the risk of cancer by up to 10–40% [53,59]. Reducing ovarian cancer mortality may be achieved by developing appropriate screening tools [58].

### 3.3. Corpus Uteri Cancer

Corpus uteri cancer, also known as endometrial cancer, became the most common gynecological malignancy in high-income countries with a number of 417,367 new cases in 2020 worldwide. It also constitutes the sixth most prevalently diagnosed cancer in women. According to GLOBOCAN 2020, Northern America, Europe, Polynesia, Australia, and New Zealand have the highest incidence rate, while most African regions and South-Central Asia were ranked with the lowest incidence rates. As it comes to incidence and mortality rates, there were 97,370 death cases in 2020, with the highest number in Northern America [13,60,61].

Obesity is the major risk factor for the development of endometrial cancer considering both its high rates and physical inactivity in high-income countries [62]. The association between BMI and the increased risk was especially strong for BMI above 27 kg/m^2^, with BMI over 30 increasing the risk from two to three times [63,64]. Other risk factors include age and conditions associated with metabolic syndrome such as diabetes mellitus, polycystic ovary syndrome, and hypertension [62]. In addition, chronic excessive exposure to estrogen may predispose women to endometrial cancer. This includes conditions such as unopposed estrogen therapy, estrogen-producing tumors, chronic anovulation, and polycystic ovary syndrome [65,66]. However, continuous estrogen and progesterone replacement therapy in menopause decreases the risk of disease [60,64]. Moreover, patients undergoing treatment with tamoxifen are at an increased risk because of its proestrogenic effects in the uterus [67].

Endometrial cancer has been classified into two types that differ in epidemiology, genetics, and also prognosis [66,68]. Type I tumors are the most common endometrial cancers that may arise from precursor lesions which are known as atypical endometrial hyperplasia. These low-grade tumors are associated with unopposed estrogen stimulation [69,70].

Type II tumors represent only 10% of the cases. Considering the high grade, they are associated with 40% of related deaths and carry a high risk of relapse and metastasis [65,68]. They are unrelated to hyperestrogenic conditions and develop from atrophic endometrium without any previous precursor lesions [62,70].

Abnormal uterine bleeding including postmenopausal bleeding, menorrhagia, or metrorrhagia is considered the most common clinical presentation of endometrial cancer [64]. Abdominal and pelvic examination plays an important role due to specific symptoms that may occur in advanced cases, such as abdominal or pelvic pain, abdominal distention, bloating, and change in bowel or bladder function [66,68,71].

### 3.4. Vaginal Cancer

Vaginal cancer is a rare gynecologic malignancy and constitutes only 1–2% of all female genital cancers, with 17,908 new cases diagnosed and 7995 deaths in 2020. The highest incidence and mortality rates were found in South-Central Asia and most African regions [72,73]. Each case should be histologically verified because most of the malignancies are secondary tumors or of metastatic origin. The careful examination should be performed to confirm the primary vaginal site of the growth and to exclude cervical, urethral, or vulvar origins [74,75].

More than 90% of the predominant histologic subtype cases in primary vaginal cancer are squamous cell carcinomas. Adenocarcinoma occurrence is estimated at 8–10% of cases, while lymphomas, sarcomas, and melanomas are very rare [75,76].

Symptoms such as irregular bleeding, odorous discharge, and, in advanced cases, pelvic pain or urinary retention may occur; however, 5–10% of patients remain asymptomatic [76].

Infection with high-risk human papillomavirus (HPV), the HPV 16 subtype in particularly, is associated with vaginal cancer development [77,78]. Additionally, chronic irritation is considered one of the main risk factors for the disease, especially in older women. High lifetime number of sexual partners, early age of the first intercourse, current smoking, history of cervical intraepithelial neoplasia (CIN), vaginal intraepithelial neoplasia, cervical carcinoma, and prior hysterectomy may also predispose women to vaginal cancer [78,79,80,81].

Regarding the high risk associated with HPV infection, prophylactic HPV vaccination should be considered as a primary prevention strategy to decrease the incidence of this disease [82]. In case of hysterectomy performed due to high-grade lesions, vault smears are recommended for secondary screening [83].

### 3.5. Vulvar Cancer

Vulvar cancer is responsible for 3–8% of all malignant neoplasms of the female genital organs. A total of 45,240 new cases were found in 2020 [13]. These rates are not high, but in the last few decades, it has been noticed that the overall incidence of vulvar cancer has increased [84,85]. It is primarily a disease of the elderly because more than half of the women who develop vulvar cancer are in their 70s [86].

More than 90% of all vulvar cancers are squamous cell carcinoma (SCC). The second most common type is melanoma. Bartholin gland carcinoma, Paget disease, basal, and glandular carcinomas, sarcomas, and metastatic tumors are rare [87].

Gynecological and rectal examination as well as palpation of the inguinal lymph nodes are usually sufficient to assess the stage of disease and establish a treatment plan. Most cases present symptomatically as a raised, palpable lump or a visible, erythematous lesion accompanied by pruritus, dysuria, pain, or bleeding [88,89]. Many vulvar cancers are initially diagnosed as inflammation, which delays diagnosis and worsens prognosis [90].

Treatment for vulvar cancer includes surgery, radiation therapy, or chemotherapy. The scope of the operation depends on the stage of the disease and the location of the lesions. Most often, radical vulvectomy with inguinal lymph nodes is performed. Inoperable cancers undergo systemic treatment or radiotherapy [90,91,92].

The risk factors for developing vulvar cancer include lichen sclerosus, vulvar intraepithelial neoplasia (VIN), many sexual partners, first sexual intercourse at young age, HPV and HIV infections, smoking, low socioeconomic status [92]. The most common causes of the most prevalent cancers of the female reproductive system can be found in Table 1 (Table 1).

## 4. Trace Elements and Female Reproductive Organs

Trace elements or metals are uniquely functional for the biochemical operation of the human body; a few of these can play a pivotal role in homeostasis, while others, particularly in excess, can induce toxic concentrations and instruct the body to shut down. These metals are found accumulating in the body’s tissues, some can be stored and then used for metabolic activity, and others would need a chelating substance to be removed. Nonetheless, these elements can indicate certain toxicity levels if consumed excessively or if they become deficient through diet or medication interference. Trace metals within the human body include iron, zinc, copper, chromium, cobalt, molybdenum, manganese, and others [98,99,100].

### 4.1. Iron

Iron, being one of the most versatile elements, has notably shown to be associated with hematological development of hemoglobin and myoglobin, and heme generation/degradation from the cytosol and mitochondrial matrix and as well as splenic tissue, respectively [101,102]. Iron also functions as a carrier of oxygen in the blood and muscles and is obtained via dietary consumption of poultry, fish, and meat [103]. Nonheme iron absorption is aided by ascorbic acid, but dietary fiber, phytates, and certain trace elements can reduce it. The efficiency with which the body absorbs iron from a given food is not indicated by food composition data [104]. Iron-deficiency anemia arises when the balance of iron intake, iron storage, and iron loss in the body is insufficient to fully support erythrocyte production. Iron deficiency anemia rarely causes death, although it has a significant impact on human health [105]. A hemolytic condition called sideroblastic anemia is characteristic of genetic anomaly indicating higher concentrations of iron deposits rendering erythrocytic morphology looking like sideroblasts [106,107,108]. X-linked sideroblastic anemia usually manifests itself during the first three decades of life, karyotyping results in defective genes located on the X chromosome (Xp11.21) [106,107,108,109].

#### Pregnancy/Fertility

As history serves, when the menstruation cycle initiates, the endometrial lining begins to shed its layers of the columnar epithelium; in retrospect to pregnancy, the glands and angiogenesis begin to enlarge [110]. A paper published in the Journal of Obstetrics and Gynaecology, 2016 has outlined significant resources relating to the iron supplementation and infusion of dextran-free preparations, while also producing better hematological responses compared to oral therapy alone [111,112,113,114,115,116]. Therefore, it has been an important tool in aiding the prevention of the iron-deficiency in pregnant women and hemorrhage-related maternal and perinatal mortality and morbidity.

While ferritin and iron stores serve as excellent reservoirs for a child-bearing mother, this is shared with the developmental fetus and increases the chances as opposed to women whose serum levels have lower values [117,118]. A detailed cohort study by Georgsen et al., 2021 has outlined interesting features relating to serum ferritin values with reduced chances of conception, with studies from Chavarro et al. finding that women who took iron supplements with a high iron content had a 70% lower risk of ovulatory infertility [119,120]. With the information presented from numerous articles, more research needs to be conducted, and one might need to ask whether ferritin correlates with ovulatory production and adherence with the endometrium for implantation.

### 4.2. Zinc

Zinc is another trace element of several important features and its significant role in the human body. Although the biochemical mechanisms that link zinc to physiologic functions have been well studied, the relationships between the biochemical pathways have not been fully established. Zinc’s role in biology can be divided into three broad functional categories: catalytic, structural, and regulatory functions [121]. In humans and animals, the primary means of maintaining a steady state of cellular zinc absorption are through the adjustments in total zinc consumption and endogenous excretion. The effects of these adjustments are evidenced by shifts in the concentration of zinc in the gastrointestinal tract [122,123,124,125].

An integrative review by Roohani, Nazanin et al. has cross-examined adults, infants, children, and pregnant and lactating women who have increased requirements for zinc and, thus, are at increased risk of zinc depletion [126,127]. Because infants and children are more prone to zinc depletion than adults, their increased requirements have increased the risk of zinc deficiency.

For pregnant women and children, the amount of zinc that is retained in their bodies after birth is added to the requirements. Moreover, for lactating women, the zinc secreted in breast milk provides additional nutrients.

During pregnancy and lactation, the physiological adjustments that occur during pregnancy and lactation can help meet the nutritional demands of women, though increased nutritional demands during pregnancy and lactation predispose women to zinc deficiency [125]. However, supplemental iron can reduce zinc absorption and limit the zinc deficiency that can be expected to occur [128,129].

Zinc deficiency is a condition that affects various organs such as the central nervous system, gastrointestinal, central nervous, immune, skeletal, and reproductive systems, and the epidermal glands; the signs of this condition vary depending on its severity [130].

Zinc deficiency or chelation of zinc disrupts the maturation of oocytes and reduces their quality, so an adequate supply of zinc is required for the oocyte to form a fertilization-competent egg. According to Garner et al., symmetric division, proliferation, and differentiation of the preimplantation embryo are all dependent on zinc availability, both during oocyte development and after fertilization [131].

Grieger, Jessica A et al. presented interesting data explaining the relationship with zinc and time to pregnancy, which displayed from 15 ± 1 weeks that lower (<7.80 µmol/L) but not higher (>12.24 µmol/L) zinc concentrations were also associated with a prolonged time to pregnancy; notwithstanding subfertility, zinc has not made any associations [23]. After controlling maternal and paternal factors, women with circulating zinc concentrations in the lowest reference range in early pregnancy took 0.6 months longer to conceive than women in the middle tertile.

In a population-based case-control study conducted in California between 1989 and 1991, the authors investigated the relationship between maternal pre-conceptional supplemental and dietary zinc intake and the risk of neural tube defects (NTDs). There were 430 NTD-affected fetuses/infants in the study, and 429 nonmalformed infants chosen at random. Mothers reported their use of vitamin, mineral, and food supplements before conception and completed a 98-item food frequency questionnaire. Increased total pre-conceptional zinc intake was linked to a lower risk of neural tube defects [132].

Zinc is essential for ovulation and the menstrual cycle. ROS imbalance affects oocyte maturation, ovulation, luteolysis, and follicle atresia. Follicogenesis, follicular atresia, and luteal regression are all influenced by OS and apoptosis. Oxidative species, in particular, has a negative impact on second meiotic division progression, decreased gonadotrophin, antisteroidogenic effects, DNA damage, and inhibited protein ATP production [133,134,135].

### 4.3. Copper

Copper is abundant in foods such as organ meats, seafood, nuts, and seeds, and it is essential for oxidative processes, energy metabolism, free-radical defense, and iron transport. According to the Adelaide SCOPE cohort, women with lower plasma copper concentrations were protected against the risk of any pregnancy complication when compared to women with high plasma copper concentrations [136], raising the possibility that copper is more important for placentation than conception; undoubtedly, more research is needed to make a more definitive association.

Female contraception is common in today’s society and reduces the risk of pregnancy, particularly in young adults. Contraception products come in multiple forms; we will only discuss intrauterine copper. Hormonal contraceptives appear to be responsible for reducing circulation levels of androgen, estradiol, progesterone, and oxytocin inhibition. Hormonal usage of contraception may change the pairing behavior of women, diminish the brain reaction to erotic stimulus expectations, and enhance the sexual jealousies of women [137]. With copper intrauterine contraception, women have endorsed good satisfaction rates and have also observed no general or psychological differences in sexual function [137,138].

In clinical practice, to improve the knowledge of kinds of sexual behavior and patterns of sexual function over life and beyond, including how sex can and cannot be affected by age, it is necessary to address and assess the psychosocial status and behavior of the patient [139]. The results of the research show that hormonal contraception may affect many elements of the sexual function of women. However, many studies on the relationship between sexual and hormonal contraceptives produce inconsistent results; thus, it can be concluded definitely that further study is necessary [138,139,140].

Carrascosa et al., 2017 aimed at the effect of copper on apoptosis and necrosis, visualized through immunofluorescence, and then determined the profile of gene expression from a 192 gene panel, linked by quantitative reverse transcription PCR with endometrial receptivity and the immune system (RT-qPCR) [141]. Out of these genes, the treatment with copper-induced changes in the expression of 129 genes (94 up- and 35 downregulated) (ultimately, intrauterine devices have modified endometrial physiology to avoid conception) was carried out. Because of the endometrial release of copper ions, copper IUDs should have extra effects on inert intrauterine devices (IUDs) [141,142]. This study has produced astonishing genetic data regarding copper and gene expression on specific markers for endometrial receptivity. Treatment with copper altered the decidualized endometrial stromal cells’ (dHESCs’) decidual gene signature, including several genes involved in endometrial development and endometrial diseases. Interestingly, copper reduces IGFBP1 levels by half, a well-known marker of decidualization. Nine of the 49 genes that are dysregulated by copper therapy are also associated with the decidualization process (*CXCL6*, *CSRP2*, *FOXO1*, *IL1R1*, *IL8*, *IL11*, *IL15*, *LIF*, and *TNFRSF11B*) [141].

The most common adverse effect of copper IUDs is increased menstrual bleeding, which may persist over time, modifying subendometrial microvascularization [143,144]. Nulliparous women who utilize copper IUDs had a removal rate ranging from 3.6 percent to 24 percent, owing to discomfort and bleeding, which may be slightly greater than in parous women [144]. We suggest that copper’s modification of the endometrial gene signature should involve numerous genes associated with endometrial illnesses and disorders, and that this disruption of the gene pattern might be the cause of these adverse effects from copper IUD [141,143,144].

Noda et al., 2012 investigated the plasma levels of pituitary and ovarian hormones that are involved in the reproductive system in Sod1^−/−^ female mice. At 22–25 weeks of age, the Sod1^−/−^ female mice had substantially lower body weights than the Sod1+/+ mice. The results revealed that a cytoplasmic deficit in CuZn-SOD led to reduced luteinization and serum P_4_ induced by increased ROS generation in eggs [145]. The authors strongly suggested that increased oxidative stress in the ovary might contribute to miscarriage in mice. Older patients in the clinic exhibit a greater risk of error. These present an unknown etiology, which prevents suitable treatments from being selected. While the origin of error remains unknown, intracellular oxidative stress can be a cause [145]. There is much more to explore in this area, and finer experimentation is needed to analyze female fecundability and female reproductive organs.

### 4.4. Fluoride

Fluoride is one of nature’s most abundant elements. Fluoride is primarily found in water. The risk of dental caries is the only known association with low fluoride intake. Fluoride is one of the few trace elements that are under privation and is undervalued when it comes to systemic health benefits or possible detriments. Because fluoride exists in drinking water in ionic form, it passes quickly through the intestinal mucosa. Fluoride is a small-molecular-weight anion that affects organisms by combining with calcium ions (Ca^2+^). It can easily pass through cell membranes and disrupt cell metabolism and function through simple diffusion. However, in recent years, some scientists have assessed and classed fluoride, which is mostly cited in the Lancet Neurology multiple times in epidemiological studies [146] as a neurotoxicant that reduces measures of intelligence in children, placing it into the same category as toxic metals (lead, methylmercury, arsenic) and polychlorinated biphenyls [147,148,149]. Excessive fluoride intake, over a long period of time, may result in a serious public health problem called fluorosis, which is characterized by dental mottling and skeletal manifestations [150,151].

Search engines such as PubMed returned only 32 published articles when searching for “(Fluoride) AND (Female Fertility),” with the vast majority relating to preclinical data on murine and other animal fecundability studies, mostly on the male fertility aspect.

Human cohorts recorded on female pregnancy only accounted for post-partum patients and checking the IQ levels of young infants and children from 3 to 16 years of age assessing their cognitive ability and behavioral traits [152,153,154,155]. Considering the lack of knowledge related to fluoride and female fertility, this gap of knowledge surely needs to be filled.

Zhou et al., 2013, amongst the few published articles on fluoride and female fertility, outlined the mechanism of sodium fluoride (NaF) on female reproductive organs such as the uterus, ovaries and their hormonal/ biochemical synergies. What they discovered was an increase in estrogen receptor alpha protein (ERα) expression in all of the NaF-treated groups, particularly the 100 mg/L groups. Increased ERα expression may result in decreased endometrial receptivity and impaired embryo implantation [156]. ERα and progesterone receptor protein, on the other hand, were upregulated in the 100 mg/L group. These findings led us to believe that the discrepancies are attributable to the fluoride ion’s lower ionic mobility [156]. These hormones are essential for oocyte development and pre-implantation of the uterine walls.

The fact that several other studies were conducted using laboratory animals that were exposed to a range of fluoride concentrations (0.1–250 mg/L in the drinking water) indicated that exposure to relatively high concentrations of fluoride resulted in adverse reproductive and developmental outcomes [157].

Therefore, it could be assumed that Zhou et al. indicated that high fluoride intake by drinking water could reduce female reproductive function by affecting steroidogenesis as well as steroid hormone receptor expression.

### 4.5. Selenium

Selenium (Se) is an essential trace element that is integrated into selenoproteins, exhibiting a variety of pleiotropic effects ranging from antioxidant and anti-inflammatory benefits to active thyroid hormone synthesis [23]. Selenium, amongst other micronutrients is often thought of as something essential; in the clinical and medical sense, it is demonstrated to be one closely related to mortality, thyroid function, and cardiovascular systems [158].

A review in The Lancet 2012 compiled at least 10 years’ worth of selenium-related input on human health and awareness in terms of its benefits and its toxicity [159]. Clinical data relating to serum or plasma selenium concentration varies among supercontinent values around the world. Australia, being one of the richest domains on the planet, and China are amongst the most deficient in certain areas of the country [160,161]. As for dietary intakes, it is suggested from a study published by Newman, 2019 and Fairweather-Tait et al., 2011 that men should consume 60 µg of selenium per day, while women should consume 53 µg per day [162,163].

Plasma and serum Se concentrations can relate to health and disease; however, when recording and reviewing published laboratory values of selenium, they seem to be similar. A study in the Journal of Ecotoxicology and Environmental Safety shows enthralling evidence comparing trace elements in both maternal serum, follicular fluid, and their association in blastocyst formation and positive correlation on embryo development [164]. Wu et al., 2020 creatively explore in vitro fertilization (IVF) couples and assess both female and male counterparts on toxic and essential trace elements, and they have reported that male seminal Se levels were found to be favorably related to pregnancy and live births [164]. Embryological development research is focusing on follicular and serum Se concentrations; follicular Se concentrations were found to be positively associated with embryo quality at the cleavage stage, whereas higher female serum Se levels were found to be significantly associated with higher probabilities of blastocyst formation and had a borderline significant association with high-quality blastocysts [164]. Maeda et al., 2019 reported multiple logistic regression studies that controlled for other metals, and other covariates revealed substantial correlations between infertility and low Se levels, therefore suggesting a protective characteristic [165].

A double-blind randomized prospective study performed on 120 female patients undergoing assisted reproductive interventions indicated that oral Se supplementation was significant in terms of obtaining good-quality embryos [165]. The suitable effect of Se on embryo development is consistent with the result that female serum concentrations were complimentary with blastocyst formation probabilities [165,166,167]. Mantovani et al., 2019 produced outstanding figures inside their “SERENA study”, a randomized, double-blind, placebo-controlled trial; this involves pregnant women diagnosed with autoimmune thyroid diseases, and the research found that taking 83 mcg of selenium per day throughout pregnancy and after delivery is safe and has a positive effect on autoantibody titer and postpartum thyroiditis recurrence [167].

The inextricable U-shaped relationship between status and Se’s health consequences must be stressed, and Se was initially known as a hazardous component [159,160,164]. Another explanation for the inverse relationship between follicular Se and blastocyst development is that long-term high Se exposure may have a negative impact on human reproduction.

IVF treatment has featured in recent years to help facilitate pregnancy and aid in fertilization for couples trying to conceive. Females’ fecundability is often associated with significant outcomes. However, credible cohort studies have shown that significant increase in Se supplementation for both male and female counterparts have improved not only the fecundability—a favorable relationship was found between female serum Se levels and embryo growth at the blastocyst stage in couples receiving IVF treatment. Furthermore, Wu et al., 2020 may have uncovered greater seminal Se levels, and lower follicular cadmium (Cd) levels were linked to a higher likelihood of pregnancy and live birth [164].

However, an extremely low Se status may cause an imbalance of antioxidants with an accumulation of reactive-oxygen species (ROS), which is often considered as a risk factor for polycystic ovarian syndrome In any event, a low Se status in the body reduces resistance to free-radical-induced damage; this may be due to its participation in the synthesis of selenoproteins as well as its antioxidant capacity [168,169].

Selenium-binding protein 1 (SBP1) is specific to ovarian autoimmunity and primary ovarian insufficiency (POI) [168]. Edassery et al., 2010 discovered antigens that were often associated with serum autoantibodies in women with idiopathic infertility and POI. SBP1 levels were found to be significantly greater in women suffering from idiopathic infertility and premature ovarian failure [170].

Following relevant data, treating dairy cows by inoculation of Se has reduced ovarian cysts, though human RCT is a must to decipher clinical and medical reasoning and observe biochemical natures of fecundability and pathology [169,171].

Pregnancy-related problems, such as pre-eclampsia, intrauterine growth restriction, gestational diabetes, and premature labor, are all influenced by placental oxidative stress [172]. As previously mentioned above, Se aids in positive embryo maturity, though a select few studies focused on trophoblastic cells being stimulated with Se to activate mitochondrial biogenesis for the reducing of ROS [168,172,173,174,175]. Bizerea et al. (2018) evaluated the link between glutathione peroxidase, a strong cytosolic enzyme that aids in controlling ROS and RNS (reactive-nitrogen species) levels, and redox balance in nearly all tissues. Selenium insufficiency has held a record for hypertensive diseases that develop in one out of every ten pregnant women, particularly during the second half of the gestation period [176].

### 4.6. Chromium

Chromium has been listed as an essential trace element in the leading literature, labeled as an important link with carbohydrate metabolism, and a lack of chromium results in reduced glucose tolerance, which works by amplifying the effect of insulin [177,178,179]. However, according to Di Bona et al., 2010, chromium is not a necessary trace element [180,181,182]. Chromium comes in the forms of Cr^6+^ compounds (man-made material) and Cr^3+^ compounds which are present in soil and plants [183], which could manifest themselves into toxicosis potential to induce lung and other forms of cancer [184]. Chromium deficiency symptoms are considered to be comparable to type 2 diabetes and cardiovascular disease [177,184,185]. Reported cases of toxicosis in females working under industrial environments and an observation of hemorrhagic patches related with chromium prevalence in the umbilical cord of mothers have shown that chromium interferes with the regulation of ovarian function in exposed women who have irregular menstruation [186,187].

Chromium has not been professionally researched as a sole contributory factor under female fertility or fecundability thus far, though animal models were used to access physiological reproductive function in male murine models [188].

A review published by Faghfoori et al., 2017 involving polycystic ovary syndrome (PCOS) showed that nutritional management to insulin resistance by supplementing chromium for therapeutic purposes can control insulin resistance and weight management, but further studies are needed to exclude contradictory indications [189]. PCOS is characterized by irregular menstruation, polycystic ovaries, hyperandrogenism, insulin resistance, and obesity, and affects 5 to 20% of women, depending on the diagnostic criteria used [190]. All of these are subdued from a magnitude of biochemical actions, some relating to insulin resistance and glucose sensitivity, making it a probable relationship for chromium use to potentially alleviate symptoms via supplementation.

Reddy et al. (2013) investigated the possible protective role of α-tocopherol in chromium-induced oxidative stress in rat female reproductive system. What was reintroduced, along with other relevant material from the same field of expertise, was the usage of the antioxidant vitamin-E, which showed substantial protective benefits [191]. Chromium administration can cause oxidative stress in the female reproductive system of rats, which could alter histoarchitecture, including atrophy of endometrial glands in the uterus, hyperplasia of the uterine epithelium, and fibrous tissue growth [191,192,193].

Short-term and long-term exposure to both trivalent and hexavalent chromium exposure exhibited a substantial reduction in the number of implantation sites and viable fetuses [194]. Females exposed to hexavalent chromium had a substantial increase in the number of resorptions. Elbetieha and Al-Hamood, 1997 also assessed the weight of the organs and highlighted an increase both in male and female reproductive organs with the ovarian organ significantly weighing more, opposed to the uterus, which weighed less [194]. A study conducted by Bataineh, Bataineh, and Daradka, 2007 outlined the levels of potassium dichromate (hexavalent chromium) in embryos and fetuses of pregnant rats exposed to chromate, which are reported to be 10-fold higher than those detected following exposure to equivalent dosages of chromium chloride (trivalent chromium) [195]. Chromium compounds have been found in experimental models to be embryotoxic and teratogenic and to impair fertility and reproductive functions, as well as to eliminate aggressiveness and sexual behavior [195,196].

### 4.7. Iodine

Iodine is an essential trace element found in fish, poultry, and seaweed and is available as a dietary supplement. Iodine is vital for human health and is used in the biosynthesis of thyroid hormones and normal functionality of the thyroid gland. Thyroid hormones are key regulators of metabolic activity because they control numerous fundamental biochemical events, such as protein synthesis and enzyme activity [197,198].

Thyroid function plays a pivotal role in human homeostasis; underactive and overactivity etiology is well documented for research and clinical uses to this day. Thyroid-stimulating hormone (TSH), also known as thyrotropin, is secreted to regulate thyroid hormone synthesis and secretion, therefore protecting the body against hypothyroidism and hyperthyroidism. TSH utilizes and reuptakes iodine and stimulates triiodothyronine (T3) and thyroxine (T4) [199]. TSH levels stay elevated in the lack of adequate iodine, resulting in goiter, an enlargement of the thyroid gland caused by the body’s effort to capture more iodine from the blood and create thyroid hormones [198].

Iodine deficiency exerts multiple adverse effects on growth and development that are preventable [200]. Fifty percent of Europe is still slightly iodine deficient, while iodine intakes in other developed nations, like the United States and Australia, have decreased in recent years by introducing iodized salt; the FDA has approved the use of potassium iodide and cuprous iodide for salt iodization [201,202]. Iodine deficiency disorders are listed under specialized criteria relating to infertility and are associated with abortions, stillbirths, and congenital anomalies [200,203], this being a tender subject for unexplained circumstances.

The main corpus for understanding iodine and its uses on human health comes under the specialty of endocrinology. Having said this, physicians try to understand the complexity of both hypo- and hyperthyroidism through screening protocols. In this study, the understanding of female fertility and iodine have been cross-matched on several occasions through in vivo and in vitro studies.

Research published in Advances in Nutrition, a peer-reviewed publication, investigates female fertility and the nutritional approach, which is gaining popularity throughout the world. Skoracka et al., 2021 have identified a potential link for a holistic approach to improving female fertility and overall fecundability for females of childbearing ambitions [204]. It has been established that the consumption of trans-fatty acids, processed carbs, and added sugars has a detrimental impact on female fertility. A diet rich in dietary fiber, omega-3 fatty acids, vegetable protein, vitamins, and minerals, on the other hand, has a beneficial influence on female fertility [205,206,207]. The Mediterranean diet has been linked to improving female fertility. There is confounding evidence around omega-3 fatty acids from oily fish and supplements have been shown to increase oocyte development and maturation, reduce the chance of anovulation, and improve embryo shape [208,209]. However, Wise et al., 2017, Mumford et al., 2018 and Chiu et al., 2017 provide details of no known association, but this typical area still needs to be explored in future studies [207,210,211]. This being mentioned, a few studies may choreograph a synopsis relating iodine and omega-3 supplementation on the development of pre-exposed mothers of hypothyroxinaemia; at least in murine models it shows something beneficial [212,213,214].

Iodine and TSH indirectly promote ovulation by stimulating follicular oocyte growth by acting on receptor-ligand binding of follicle-stimulating hormone (FSH) [215]. Estrogen can influence iodine absorption by the ovaries. Estradiol promotes proliferation while suppressing sodium-iodine symporter gene expression, which can explain iodine accumulation in the walls of large Graafian follicles, followed by a shift to the follicular fluid [216,217,218].

An interesting comparative study by Johnson et al., 2005 highlighted a potential step forward to minimize infertility via the mechanisms of facilitated lipiodol into the uterus of animal models. Upon reflection, antigen-presenting cells may control the formation and maintenance of an implanted embryo that is alien to the endometrium [219]. However, compelling evidence from using high doses of iodine, whether in concentrated or large amounts, may interfere by causing edema and hemorrhage in the lamina propria of the uterine tissue [218,219,220]. There is still much to be explored, and with today’s advances and a better understanding of the topic, there is potential to underpin fertility and iodine usages.

Abel et al., 2020 published a cohort paper involving 78,318 pregnancies that revealed insufficient iodine intake was associated with reduced fetal growth and increased risk of preeclampsia in this “mild-to-moderately iodine-deficient pregnant population”. There were also indications of an increased risk of preterm delivery and subfecundity [221]. This cohort revealed an assessment of dietary intake of iodine within their population, measuring urine iodine concentration and surveying foods/drink intake regularly without the use of iodine supplementation. Here they recorded values being under/below the WHO recommended values (median UIC ≥ 150 μg/L for pregnant women and median ≥ 100 μg/L for nonpregnant) [222], which become apparent with all necessary findings. From the accumulative data, ~100 μg/day or lower increased the prevalence of pre-eclampsia and preterm delivery, but not with early preterm delivery or intrauterine death [223]. A comparison was done and showed values as low as 50 μg/day increased the risks of pre-eclampsia.

It has been suggested that sufficient amounts of iodine in childbearing age is recommended, not only for appropriate fecundability but for the overall growth, and both pre- and post-partum developments of children were closely looked into.

### 4.8. Manganese

Manganese is found in foods naturally and is a cofactor for many enzymes, including manganese superoxide dismutase, arginase, and pyruvate carboxylase [222,224,225]. Manganese is involved in the metabolism of amino acids, cholesterol, glucose, and carbohydrates; reactive oxygen species scavenging; bone formation; reproduction; and immunological response [226,227]. Manganese, in combination with vitamin K, also aids in blood coagulation and hemostasis.

Manganese status can be perplexing because it is not regularly evaluated in clinical practice. Manganese values in whole blood vary from 4 to 15 mcg/L [224], though in clinical biochemistry, manganese comes under the bone profile for its mineral status.

Having extracted information from Pubmed and Google Scholar search engines, there is some limited information published; then again, manganese is typically grouped with other minerals as part of a screening regime for disease-specific associations.

The information drawn in terms of reproduction and fertility has typically been formulated for experimental use and analysis, particularly assessing methods of inhalation of manganese dichloride in rat models, though conclusions are promising to have negative effects on reprotoxicity [228]. This having been said, more animals’ studies further enhance the association on reproductive hormones, gonadotropin-releasing hormone (GnRH), and FSH expression significant increases in breeder broiler hens [229]. The results of serum Mn concentration have strengthened eggshell with no deviation from normal breeding. With this relevant information conducted, the association in human models could perpetuate and potentially strengthen oocyte growth and maturity. The relationships with Mn and hormonal activity may strengthen and compensate for balance [229].

Celine Faure et al. may have stumbled across a potential genetic link with infertility, a case-control study conducted to cross-examine antioxidant gene polymorphisms; superoxide dimutase-2 and nitric oxidize synthase enzymes for idiopathic infertility [230]. The identified gene, G-eNOS allele (rs1799983), expression has increased significantly; therefore, there is a risk of infertility both in men and women [231,232]. Based on this hypothesis, the susceptibility of oxidative stress should be explored more thoroughly for females and should question the genetic variability and determine whether it is population based, a hereditary linkage, or something else: can this potentially be inhibited with sufficient trace element blood concentration or influenced by manganese?

Skogheim et al., 2021 conducted an in-depth investigation into whether maternal levels of hazardous metals and critical elements assessed in mid-pregnancy, both separately and in combination, were linked to a juvenile diagnosis of attention-deficit/hyperactivity disorder (ADHD) or autism spectrum disorder (ASD). The current study’s findings indicate many links between metal and element levels during pregnancy and ASD and ADHD in children [233]. Manganese becomes a notable metal amongst the results of this study; suggesting that neurodevelopment may potentially have detrimental consequences in population levels of these chemicals. Because the primary effects of metals and ions on ASD and ADHD were comparable, it might be that both illnesses share some neurochemical and neurodevelopmental paths [233].

### 4.9. Aluminum

Aluminum is considered as a trace element, but it presents no biological component necessary for human activity. As it stands, it is used commercially, in welding and car manufacturing, and in cans for everyday consumables. Just like other trace metals, there are toxic and nontoxic components to aluminum; for instance, blood serum screening for aluminum ranges from 1–3 µg/L and >50 µg/L may indicate toxicity [234]. Aluminium exposure from food, water, and dermal contact (e.g., antiperspirants and sunscreen lotion) is minimal. Aluminium intake is highest from aluminum-containing medications, such as antacids and antidiarrheal and antiulcerative medications. Aluminium accumulates in the skeleton and lungs, but excess amounts have the greatest impact on the brain and nervous system [235,236].

Hirata-Koizumi et al., 2011 composed a two-generation murine study using aluminum ammonium sulfate, and the results are in keeping with weight loss which can delay growth in pre-pubescent mice [237]. Rats with delayed vaginal opening demonstrated normal reproductive ability and result in the current investigation. Furthermore, there were no impacts on estrous cyclicity, weight, or the histopathology of reproductive organs in weanlings or adults.

A study published in the J Trace Elem Med Bio highlighted fine ultrastructural details of cellular components from myometrial and endometrium cells, and in the cells of the ovary (internal theca and granulosa cells). Lysosomes were extracted, and the analysis compared with other tissues shows crystallized fragments inside the lysosomes as part of their cellular role by phagocytosing aluminum ready for disposal [238]. Here a suggestion was made to assess endocrine function and female fertility with aluminum exposure. Overall, there is much to be examined and explored to determine aluminum’s role in human health and female fertility. Table 2 presents a summary table regarding the concentration of trace elements and their physiological significance in the female reproductive system.

## 5. Discussion

In this review, we aimed to summarize the state of current knowledge on trace metals concentration and its association with the fertility and carcinogenesis of the organs of the female reproductive system (Table 2). It has been shown that these elements may indicate certain toxicity levels if consumed excessively or may become deficient through diet or medication interference.

Iron supplementation and infusion of dextran-free preparations played an important role in aiding the prevention of the iron deficiency in pregnant women and hemorrhage-related maternal and perinatal mortality and morbidity. Additionally, women who took iron supplements with a high iron content had a 70% lower risk of ovulatory infertility.

When it comes to zinc, its deficiency or chelation disrupts maturation and reduces oocyte quality, so its proper supplementation is crucial for the oocyte to form a fertilization-competent egg. Moreover, symmetric division, proliferation, and differentiation of the preimplantation embryo depend on zinc availability. Lower zinc concentrations were also associated with a longer time to pregnancy. Additionally, increased total preconceptional zinc intake was linked to a lower risk of neural tube defects among infants.

A correlation between follicular Se concentrations and embryo quality at the cleavage stage was found. Higher female serum Se levels were associated with higher probabilities of blastocyst formation and also with high-quality blastocysts. In the case of IVF treatment, an increase in Se supplementation for both male and female counterparts have improved both fecundability and embryo growth at the blastocyst stage.

Chromium administration can cause oxidative stress in the female reproductive system of rats, leading to many alterations in the histoarchitecture of endometrial glands, uterine epithelium, and fibrous tissue. Unfortunately, trivalent and hexavalent chromium exposure reduced the number of implantation sites and viable fetuses. In experimental models, chromium compounds were found as embryotoxic and teratogenic.

Insufficiency in iodine intake was associated with reduced fetal growth and increased risk of pre-eclampsia, preterm delivery, and subfecundity in the pregnant population.

A relationship between high fluoride intake and reduced female reproductive function was indicated. However, there is still an urgent need to explore this area due to a scarcity of literature sources relating to fluoride, aluminum, and manganese’s role on human health and female fertility.

Except for proper trace elements homeostasis, what is crucial in women’s health is vitamin balance, and the number of research regarding vitamin disbalances and nutraceutical supplementation to counteract them is continually increasing. As an example, Paul et al. showed that synergistic supplementation of myo-inositol and D-chiro-inositol might be helpful in the case of the polycystic ovarian syndrome as well as metabolic syndrome [253]. Further, numerous environmental toxicants might disrupt maternal and fetal homeostasis, leading to impaired pregnancy or fetal development, and they might act as a trigger for numerous disorders and malignancies of the female reproductive system. Such compounds as bisphenol A, herbicides, organophosphate pesticides, and particularly organochlorine compounds (e.g., dioxins, chlorinated pesticides, polychlorinated biphenyls) might seriously damage fertility [254]. Sofo et al. showed that exposure to 2,3,7,8-Tetrachlorodibenzo-*p*-dioxin (TCDD) might be associated with the pathogenesis of endometriosis; the role of other endocrine disrupters should be further investigated in the future studies [255]. Environmental toxins and chemicals might also significantly contribute to the onset of numerous cancers of the female reproductive organs, including endometrial cancer, uterine cancer, or cervical cancer [256,257,258].

## 6. Conclusions

Trace elements homeostasis maintains the concentrations within the physiological values enabling proper functioning of the organism. The maintainance of proper values of chosen micro- and macronutrients during pregnancy seems to be important for the proper development and growth of the fetus during pregnancy. This is because selected trace elements constitute the components of chosen regulatory enzymes as well as hormones that enable either differentiation or division of fetal cells during the further development of a fetus. Further, exposure to selected toxic elements could be harmful to both the mother and the fetus and is considered to be associated with impaired oocyte maturation, ovulation, and even fertilization; thus, it might impair further fetal development. Regarding further research, studies on trace elements concentrations especially in maternal serum, umbilical cord blood, or amniotic fluid are highly recommended for the understanding of maternal health as well as the developmental status of the fetus during the pregnancy period; the indication of reference values of chosen trace elements in the abovementioned environments would also be highly desirable. Further, studies should also focus on such aspects as interactions between selected micro- and macronutrients during the pregnancy period along with the establishment of recommendations regarding micro- and macronutrients supplementation, especially during pregnancy. Another potential aspect that could possibly be examined more deeply by the researchers is the hypothesis that some of the chosen elements, e.g., copper (Cu), could act as potential biomarkers of inflammatory response; an indication of other potential trace elements, along with their concentrations (and interactions between one another) that could induce an inflammatory response, might reduce the number of potential complications during pregnancy. As it comes to the research on female fertility, studies that would examine the relationship between the circulating micro- and macronutrients along with any toxic elements and female fertility are highly recommended. The examination of serum trace elements concentrations in patients with cervical, ovarian, corpus uteri, vaginal, and vulvar cancers as well as amongst healthy controls would also provide us with insight into a better understanding of the pathogenesis of the aforementioned malignancies. Such studies could also potentially indicate some kind of prevention or even additional treatment strategies amongst such patients.

## Figures and Tables

**Table 1:** The most common causes of the female reproductive system cancers.

Type of Cancer	Causes	References
Cervical	Human papillomavirus (HPV) infection	[51]
	Tobacco smoke	[93]
	Oral contraception	[93]
Ovarian	BRCA Gene Mutation	[94]
	Postmenopausal hormone-replacement therapy	[95]
	Tobacco smoke	[95]
Corpus uteri	Obesity	[63]
	Estrogen hormone replacement therapy	[60,65]
	Estrogen-producing tumors	[66]
	Tamoxifen	[62,64]
	Lynch syndrome	[62,96]
Vaginal	Human papillomavirus type 16	[77,78]
	Smoking	[79]
	Chronic irritation	[79]
	Vaginal and cervical intraepithelial neoplasia (CIN)	[80,81]
	Cervical carcinoma	[80]
Vulvar	Human papillomavirus (HPV) infection	[97]
	Chronic inflammation	[87]
	Autoimmune-related processes	[87]

**Table 2:** Summary table regarding the concentration of trace elements and its significance in female reproductive system physiology.

Element	Ref.	Physiological Significance	Reference Ranges (SI Units)	Reference Ranges (Pregnancy)	Toxicity	Serum/Plasma/Urine2	Essential for Female Repr. Sytem	Contraindications	Factor of Influence
Iron [Fe]	[239]	Iron is required for numerous crucial cell processes such as DNA synthesis, energy production, and proper functioning of nuclei. It is a crucial component of hemoglobin and myoglobin, involved in hematopoiesis, significant in the formation and maturation of red blood cells, responsible for oxygen transport	10.7–26.9 μmol/L			Serum	Y		++
Hemoglobin (Hb)	[240]	Normal physiological function and prevention of anemia. Advanced allosteric protein is the transportation of oxygen and carbon dioxide between the lung and the tissues	7.45–9.30 mmol/L		Micro-/Macrocytic RBC abnormalities, contributing factors will ascertain relevant pathologies.	Whole Blood	Y	Various types of anaemias and haematological pathologies and metabolism.	++
TIBC	[241]	Considered as a measure of transferrin (Tf) concentration in serum or plasma. Diagnosis for IDA.	44.8–71.6 μmol/L			Serum	Y		++
Ferritin	[242]	Storage protein of iron and acts on the homeostasis and sequesteration	15–200 μg/L			Plasma	Y	liver disease, rheumatoid arthritis, other inflammatory conditions or hyperthyroidism	+++
Transferrin	[243]	Blood-plasma glycoprotein, which plays a central role in iron metabolism and is responsible for ferric-ion delivery	2.0–3.8 g/L			Serum	Y		++
Zinc	[244]	Biological processes, as a structural, catalytic, and intracellular and intercellular signaling component	7.7–23.0 μmol/L	≤23.0 μmol/L ***	≥23.0 μmol/L ***	Serum	Y	Nutritional deficiency from breast feeding, synergic interuption from iron supplementation, and prolonged pregnacy, subfertility.	+++
Copper	[245,246]	As a catalytic cofactor in the redox chemistry of enzymes, mitochondrial respiration, iron absorption, free radical scavenging, and elastin cross-linking, it plays a critical role in cell physiology	11.0–22.0 μmol/L	≤22.0 μmol/L ***	≥23.0 μmol/L ***	Serum	N/A	Contraception elements to reduce risks of pregnancy, hormonal amplitude, mood swings and induced cyclical bleeding. Anaemia, leukopenia, bone abnormalities, decreased pigmentation of skin and hair, neurological derangement	++
Fluoride	[157]	Accumulates in the body’s hard tissues and is known to serve a crucial role in the mineralization of bone and teeth	1.0–100.0 mg/L	N/A	≥100.0 mg/L ***	Urine	N	Child assessment for neurocognitive dysfunction, potential endometrial and impaired embryo implantation.	++
Selenium	[161]	Antioxidant and promoter for active thyroid hormone synthesis	0.74–2.97 μmol/L	≤2.97 μmol/L ***	≥300 μmol/L ***	Whole Blood	Y	Pre-eclampsia, intrauterine growth restriction, gestational diabetes, and premature labour stem from deficiency. Hypertensive diseases in pregnancy.	+++
Chromium	[247]	At the molecular level, chromium may have a function in maintaining normal glucose and lipid metabolism. In response to an insulin-mediated chromic ion flow, the oligopeptide chromodulin binds chromic ions, and the metal-saturated oligopeptide can attach to an insulin-stimulated insulin receptor, activating the receptor’s tyrosine kinase activity	13.4–538.6 nmol/L	N/A	≥550 nmol/L ***	Whole Blood	N	Toxicosis potential to induce lung and other forms of cancer, can aid in insulin resistance.	+
Iodine	[223,248,249,250,251]	Centrally around thyroid metabolism and function, crucial for the synthesis of thyroid hormones	100–199 μg/L *	150–249 μg/L **	>500 μg/L considered excessive in pregnant women	Urine	Y	Hypo-/ Hyperthyroidism. Reduced Iodine levels may induce pre-eclampsia. Miscarriage, stillbirth, preterm delivery and fetal congenital abnormalities and pappilar cancer	++
Manganese	[226,252]	Involved in the creation and activation of several enzymes, as well as the control of glucose and lipid metabolism	182–218 nmol/L	>218 nmol/L ***	Mn shortage and intoxication have been linked to negative metabolic and neuropsychiatric consequences. The prevalence of metabolic illnesses, such as type 2 diabetes mellitus (T2DM), obesity, insulin resistance, atherosclerosis, hyperlipidemia, nonalcoholic fatty liver disease (NAFLD), and hepatic steatosis, has risen substantially in recent decades	Whole Blood	N/A, further studies needed.	Mn is a hazardous trace element as well as a vital trace element involved in human health and development. Adverse metabolic and neuropsychiatric effects	++
Aluminum	[235,236,237,238]	N/A, considered as industrial material and no biological significance	1–3 µg/L	N/A	>50 µg/L	Serum/Whole Blood	N/A	N/A, further studies required.	N/A

Key: Minimum—‘+’, Moderate—‘++’, Highly—‘+++’; * Normal—median values for children and adults, ** median values for pregnant women [246], *** Suggestive.
